# LncRNA SNHG7 promotes cardiac remodeling by upregulating ROCK1 via sponging miR-34-5p

**DOI:** 10.18632/aging.103269

**Published:** 2020-06-06

**Authors:** Jie Wang, Shouwen Zhang, Xinhua Li, Maolei Gong

**Affiliations:** 1 Department of Cardiac Intervention, Linyi People’s Hospital, Linyi 276000, Shandong, China; 2 Department of Critical Care Medicine, Aerospace Center Hospital, Haidian, 100049, Beijing, China; 3 Department of Critical Medicine, Aerospace Center Hospital, Peking University School of Clinical Medicine, Beijing 100049, China

**Keywords:** LncRNA SNHG7, cardiac remodeling, miR-34-5p, myocardial infarction

## Abstract

Previous studies have shown that lncRNA small nuclear RNA host gene 7 (lncRNA SNHG7) played an important role in cancer progression. However, the role of lncRNA SNHG7 in cardiac fibrosis is still poorly understood. In this study, the results of quantitative real time polymerase chain reaction (qRT-PCR) analysis showed that lncRNA SNHG7 was over expressed in the infarcted and peri-infarcted area in the left ventricle after MI in mice. Western blot analysis showed that knockdown of SNHG7 decreased the expression of collagen type 1 (Col1)and α-smooth muscle actin (α-SMA). Echocardiographic study suggested that inhibition of SNHG7 improved cardiac function after MI in mice. Luciferase assay indicated SNHG7 could act as a competing endogenous RNA (ceRNA) by sponging miR-34-5p. The MTT cell proliferation assay and 5-ethynyl-2’-deoxyuridine (EdU) labelling assay revealed that co-transfection of SNHG7 and miR-34-5p inhibited cell viability and proliferation of cardiac fibroblasts (CF). All the results indicated that lncRNA SNHG7 could promote cardiac fibrosis via targeting miR-34-5p through acting as a ceRNA in mice after MI. Silencing of SNHG7 could attenuate deposition of collagens and improve cardiac function. miR-34-5p could suppress the fibrogenesis of CF by targeting ROCK1 and abolish SNHG7-induced CF proliferation and fibroblast-to-myofibroblast transition.

## INTRODUCTION

Myocardial infarction (MI) is the most common event in cardiovascular diseases, accounting for the death of millions of patients within the past five years. The pathogenesis of MI is associated with various factors, such as high fat diet, diabetes, hypertension and smoking. Cardiac fibrosis (CF), also known as cardiac remodeling, has been closely related to arrhythmia, cardiac dysfunction and even sudden cardiac death. It is the pathological outcome of myocardial infarction at a certain stage, and also an important indicator of ventricular remodeling. CF is a complicated montage of various mechanisms involving many factors, including transforming growth factor-β (TGF-β), the key regulator in the pathogenesis of CF. And TGF-β/Smads signaling pathway is mainly responsible for the property ofTGF-β as a cytokine with many kinds of biologic activity. The Rho-associated, coiled-coil domain containing protein kinases (ROCK1), a regulator involved in the TGF-β/Smads signaling pathway, encodes a protein serine/threonine kinase that will be activated when bound to the GTP-bound form of Rho. Previous studies have demonstrated that ROCK1 is involved in the pathogenesis of many diseases. Upregulation of ROCK1 has been demonstrated to mediate the proliferation and metastasis of osteosarcoma cells [1]. In addition, it has been reported that ROCK1 plays an important role in pathogenesis of various tumors. Activation of ROCK1 signaling pathway has been proven to promote invasion and metastasis of pancreatic cancer cells [1] and ROCK1 may inhibit the PTEN/FAK pathway to promote the development of non-small-cell lung cancer [2]. ROCK1 is also a critical regulator of Beclin1-mediated autophagy during metabolic stress [3]. ROCK1 can also suppress the migration of inflammatory cells by regulating PTEN phosphorylation and stability [4] and it is also a critical regulator of stress erythropoiesis and fibrosis [5–10]. However, its upstream regulatory mechanism remains elusive.

More than 80% of the human genome is transcribed with only less than 2% is coding for proteins. This means that most DNA sequences are transcribed into non-protein encoding RNAs, among which, the short non-coding RNAs, such as microRNAs, have been extensively studied as potential biomarkers and therapeutic targets. Another kind of non-coding RNA, called long-chain noncoding RNA (lncRNAs), with a length of more than 200 nucleotides, has gradually come to the forefront of attention among researchers. Compared with microRNA, the gene regulation mechanism of lncRNAs is relatively more complex, involving the activation and inhibition of gene expression, as well as the regulation of chromatin structure [11–13]. Many studies have found that lncRNAs play an important role in the process of growth and development of animals and human [14–16]. Moreover, attention has been increasingly paid to the role of lncRNAs as novel biomarkers and therapeutic targets in a variety of malignant tumors [17]. Many lncRNAs has been reported to play vital roles in the proliferation, invasion and metastasis of cancer cells [18–21]. Previous studies have indicated that lncRNA SNHG7 contributed to the proliferation and metastasis of colorectal cancer and suppressed the apoptosis of gastric cancer cells [22, 23]. Besides, lncRNA SNHG7 has been found to promote tumorigenesis of breast cancer cells by activating epithelial-mesenchymal transition (EMT) and Notch-1 signaling pathway [24] as well as stabilize SDA1 domain containing 1 (SDAD1) mRNA and induce cardiac hypertrophy. But the role of lncRNA SNHG7 in cardiac remodeling caused by myocardial ischemic injury remains unknown.

MicroRNA (miRNA)s are a large group of small single-stranded regulatory RNAs that are approximately 18 to 24 nucleotides in length and encoded in animal, plant and virus genomes and they are highly conserved in evolution. They can inhibit translation and/or lead to the degradation of target mRNAs by binding to the 3 ‘- untranslated region (3’ - UTR) of target genes. In recent years, studies have confirmed that miRNAs, through regulating gene expression at post-transcriptional level, participates in embryonic development and cell proliferation, differentiation and apoptosis and are closely related to the occurrence and development of tumor, heart diseases and other diseases. miR-34-5p, a member of the miR-34 family, has been reported to negatively regulate life span [25]. But the role of miR-34-5P in heart diseases has not been scarcely described.

The findings of this study were as follows: miR-34-5p had cardio-protective effect and could attenuate cardiac fibrosis by targeting ROCK1 pathway; lncRNA SNHG7 was upregulated in mice at 4 weeks after MI; overexpression of SNHG7 inhibited expression of miR-34-5p and promoted proliferation and fibroblast-to-myofibroblast transition and silencing of SNHG7 abolished cardiac remodeling and improved cardiac function.

## RESULTS

### Inhibition of lncRNA SNHG7 attenuated cardiac remodeling induced by ischemic injury in mice.

**Figure 1:**
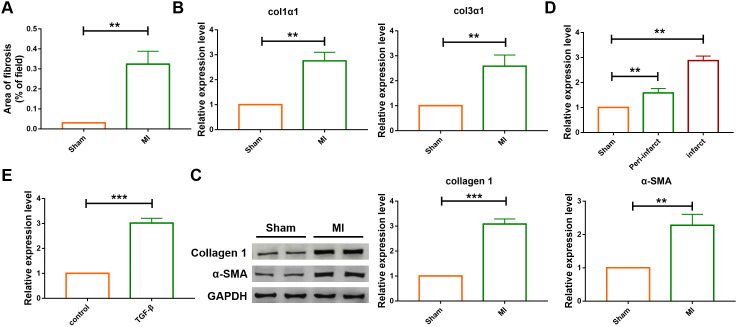
**The differential expression of SNHG7 in cardiac tissues and cardiac fibroblast.** (**A**) Quantification of the total fibrotic area using Image-J. Fibrosis areas of sham-operated group and MI group were detected by Masson staining. Data was presented as mean ± SEM; two-tailed *t* test was used for the statistical analysis. n=7 mice per group. (**B**) mRNA expression of collagen 1α1 and collagen 3α1 were measured by qRT-PCR; GAPDH mRNA served as an internal control. Data was presented as mean ± SEM; two-tailed *t* test was used for the statistical analysis. n=6 mice per group. (**C**) Protein levels of collagen 1 and α-SMA were measured by western blotting analysis; GAPDH served as an internal control. Data was presented as mean ± SEM; two-tailed *t* test was used for the statistical analysis. n=6 mice per group. (**D**) qRT-PCR analysis showing upregulation of lncRNA SNHG7 in the peri-infarcted and infarcted areas of left ventricle of mice after MI. Data was presented mean ± SEM; two-tailed *t* test was used for the statistical analysis. n=5 mice per group. (**E**) qRT-PCR analysis showing elevation of lncRNA SNHG7 in cardiac fibroblasts after treatment with TGF-β1 (10 ng/mL) for 24h. Data was presented as mean ± SEM; two-tailed *t* test was used for the statistical analysis. n=5 independent cell cultures. **P<0.05, ***P<0.001.

A four-week chronic myocardial infarction murine model was built to evaluate the effect of lncRNA SNHG7 in cardiac remodeling., The area of cardiac fibrosis was found to be increased in MI group as compared with that of the sham-operated group, as shown in Figure 1A. The result of qRT-PCR analysis also indicated that the expression of mRNA of Col 1a1 and Col 3a1 were upregulated in the ventricular tissue of MI mice (Figure 1B). Furthermore, the protein levels of collagen and α-SMA were examined using western blotting analysis (Figure 1C). Compared with the sham-operated group, the expression level of collagen and α-SMA were upregulated in mice suffering from MI. All the above data indicated the successful construction of the MI model. Then the mRNA level of lncRNA SNHG7 was determined in the cardiac tissue, cardiomyocytes and cardiac fibroblasts using qRT-PCR analysis. lncRNA level was increased both in the infarct and peri-infarct tissue as well as in fibroblasts in mice after MI, as shown in Figure 1D, 1E.

**Figure 2:**
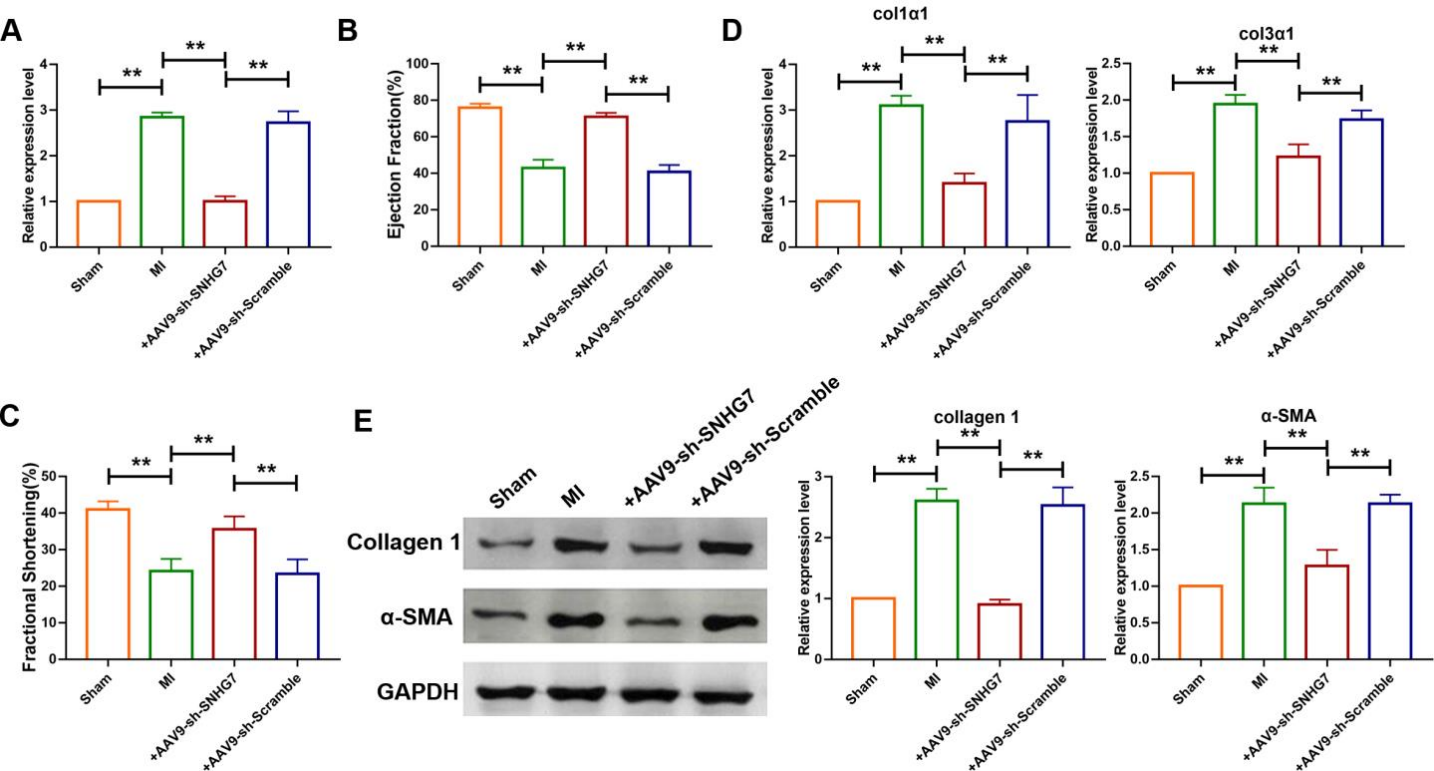
**Silencing of SNHG7 inhibited cardiac remodeling after MI.** (**A**) qRT-PCR analysis showing that AAV9-sh-SNHG7 reversed the up-regulation of SNHG7 in MI mice; GAPDH mRNA served as an internal control, and AAV9-sh-scramble served as an additional control. Data was presented as mean ± SEM; one-way ANOVA was used for the statistical analysis. n=6 mice per group. (**B**, **C**) 4 weeks after MI, echocardiographic findings showed that the silencing of SNHG7 improved ejection fraction (EF) and fraction shortening (FS). Data was presented as mean ± SEM; one-way ANOVA was used for the statistical analysis. n=12 mice per group. (**D**) mRNA expression of collagen 1α1 and collagen 3α1 were measured by qRT-PCR; GAPDH mRNA served as an internal control. Data was presented as mean ± SEM; one-way ANOVA was used for the statistical analysis. n=10 mice per group. (**E**) Western blotting analysis showing elevation of protein levels of collagen 1 and α-SMA in peri-infarcted tissue of left ventricle of mice after MI. Data was presented mean ± SEM; one-way ANOVA was used for the statistical analysis. n=5 mice per group. **P<0.05.

To explore the role of lncRNA SNHG7 in pathogenesis of MI, adenovirus carrying a SNHG7-specific short-hairpin RNA (AAV9-shSNHG7) was constructed to suppress the expression of lncRNA SNHG7 in vivo. The virus was intravenously injected via tail 1 week before the MI surgery and qRT-PCR analysis showed that the expression of lncRNA SNHG7 was significantly downregulated in mice administered with the injection of AAV9-shSNHG7 as compared with that of the MI group (Figure 2A). However, the downregulation was not observed in AAV9-scramble group. Echocardiographic findings revealed the recovery of ejection fraction (EF) and fraction shortening (FS) after the inhibition of lncRNA SNHG7 in MI mice (Figure 2B, 2C), suggesting the cardioprotective effect of lncRNA SNHG7 silencing. The expression of mRNA and protein level of collagen were also determined using western blotting and qRT-PCR analysis and the results showed that inhibition of lncRNA SNHG7 significantly diminished the expression of collagens as well as α-SMA(Figure 2D, 2E). These data demonstrated that lncRNA SNHG7 could promote cardiac remodeling after MI and silencing of SNHG7 could significantly attenuate the collagen deposition and cardiac remodeling. (Figures 1 and 2)

### Overexpression of LncRNA SNHG7 accelerated cardiac fibrosis by acting as a ceRNA sponging miR-34-5p

**Figure 3:**
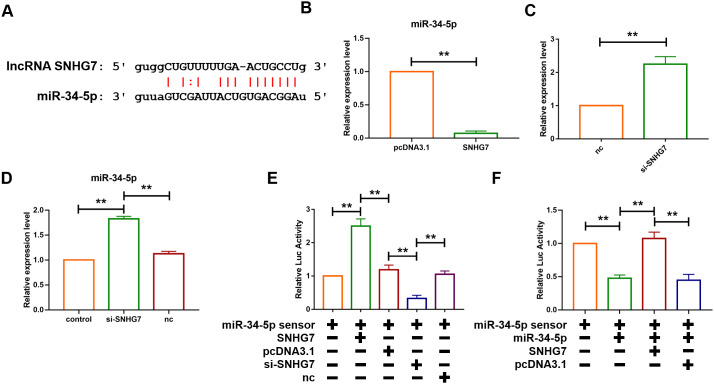
**lcnRNA SNHG7 acted as a ceRNA and sponged miR-34-5p.** (**A**) The predicted binding sites of SNHG7 and miR-34-5p. (**B**) Forced expression of SNHG7 with a SNHG7 expression plasmid inhibited the expression of miR-34-5p in cardiac fibroblasts. U6 served as an internal control. Data was presented as mean ± SEM; two-tailed *t* test was used for the statistical analysis. n=5 independent cell cultures. (**C**) Knockdown of SNHG7 by its siRNA increased the expression of miR-34-5p in cardiac fibroblasts. U6 served as an internal control. Data was presented mean ± SEM; two-tailed *t* test was used for the statistical analysis. n=5 independent cell cultures. (**D**) Knockdown of SNHG7 increased the expression of miR-34-5p in normal mice. U6 served as an internal control. Data was presented mean ± SEM; one-way ANOVA was used for the statistical analysis. n=6 mice per group. (**E**) SNHG7 binds to miR-34-5p and inhibits its activity. Cardiac fibroblasts were co-transfected with the miR-34-5p sensor and SNHG7 or si-SNHG7 and its corresponding scrambled form, and luciferase activity was detected. Data was presented as mean ± SEM; one-way ANOVA was used for the statistical analysis. n=5 independent cell cultures. (**F**) Cardiac fibroblasts were co-transfected with the miR-34-5p sensor and miR-34-5p or SNHG7 and its corresponding scrambled form, and luciferase activity was determined. Data was presented as mean ± SEM; one-way ANOVA was used for the statistical analysis. n=5 independent cell cultures. **P<0.05.

The function of lncRNAs as a ceRNA has been widely reported. To further evaluate the role of SNHG7, the starbase database (http://starbase.sysu.edu.cn/), a bioinformatics prediction website, was utilized to predict the target of SNHG7 and it was found that there are binding sites of SNHG7 on the sequence of miR-34-5p (Figure 3A). Furthermore, the loss- and gain-of-function experiment was performed to reveal the relationship between SNHG7 and miR-34-5p. The plasmid of SNHG7 was transfected into cardiac fibroblasts to overexpress lncRNA SNHG7. Overexpression of SNHG7 significantly inhibited the expression level of miR-34-5p, as compared with the pcDNA group (Figure 3B). Moreover, RNA interference experiment showed that knockdown of SNHG7 by si-RNA elevated the expression level of miR-34-5p (Figure 3C). Meanwhile, it was revealed that inhibition of SNHG7 in mice could also lead to upregulation of miR-34-5p (Figure 3D). To evaluate the targeting correlation between SNHG7 and miR-34-5p, miR-34-5p sensor luciferase reporter vector with a perfect miR-34-5p binding site containing the 3’UTR of the luciferase reporter gene was constructed. As shown in Figure 3D, the luciferase activity of miR-34-5p sensor was enhanced in cardiac fibroblasts after transfection of SNHG7, compared with the pcDNA group (Figure 3E). In contrast, SNHG7 silencing significantly reduced the luciferase activity of miR-34-5p. Furthermore, forced expression of miR-34-5p suppressed the luciferase activity of sensor and co-transfection of miR-34-5p and SNHG7 alleviated that effect (Figure 3F). Binding of SNHG7 to miR-34-5p was also detected by directly constructing luciferase reporter vector containing SNHG7 fragment with the normal or mutated binding sites for miR-34-5p. miR-34-5p inhibited the luciferase activity of the wild-type SNHG7 vector and had no effect on mutated SNHG7 vector. However, the mutated miR-34-5p had no effect on both vectors. These data indicated that lncRNA SNHG7 can directly target miR-34-5p as a ceRNA (Figure 3).

**Figure 4:**
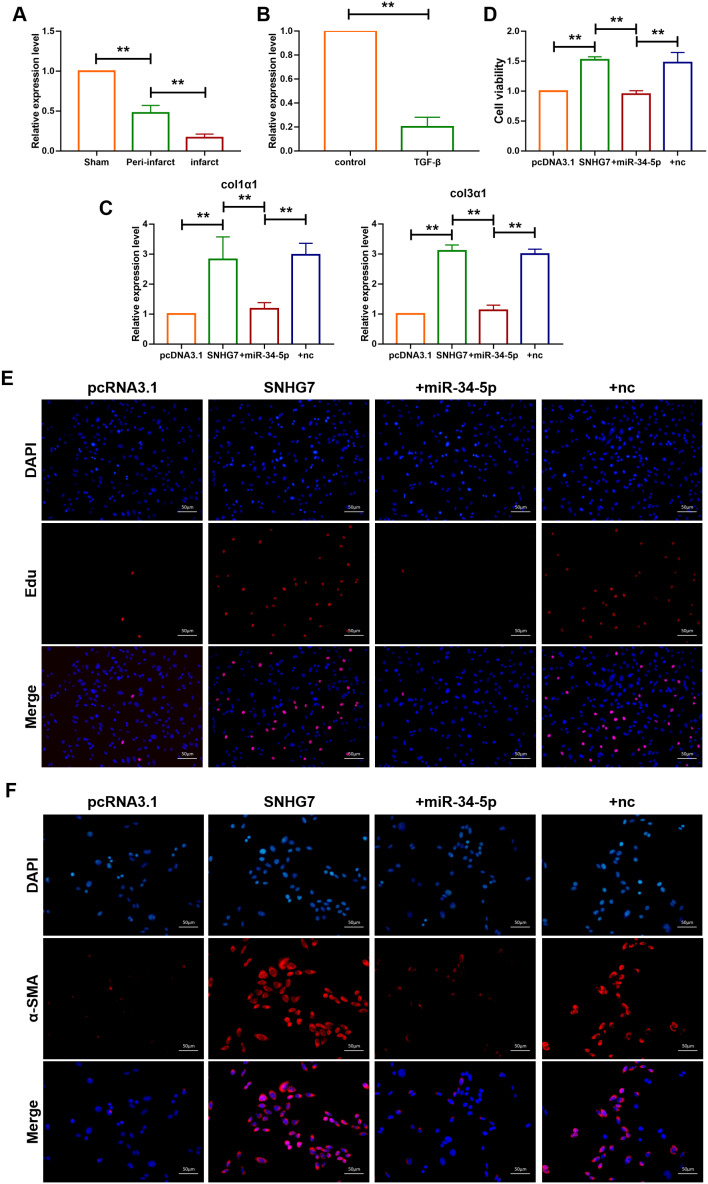
**lncRNA SNHG7 promoted cardiac fibrosis by targeting miR-34-5p.** (**A**) qRT-PCR analysis showing downregulation of miR-34-5p in the peri-infarcted and infarcted areas of left ventricle of mice after MI. U6 served as an internal control. Data was presented as mean ± SEM; two-tailed *t* test was used for the statistical analysis. n=5 mice per group. (**B**) qRT-PCR analysis showing reduction of miR-34-5p in cardiac fibroblasts after treatment with TGF-β1 (10 ng/mL) for 24h. Data was presented as mean ± SEM; two-tailed *t* test was used for the statistical analysis. n=5 independent cell cultures. (**C**) mRNA expression of collagen 1α1 and collagen 3α1 were measured by qRT-PCR. Forced expression of SNHG7 (1 μg/mL) in cardiac fibroblasts increased mRNA expression levels of collagen 1α1 and collagen 3α1, which were reversed by miR-34-5p overexpression; GAPDH mRNA served as an internal control. Data was presented as mean ± SEM; one-way ANOVA was used for the statistical analysis. n=6 mice per group. (**D**) MTT assay for the assessment of cell viability. Transfection of SNHG7 with or without miR-34-5p in normal cardiac fibroblasts. Data was presented as mean ± SEM; two-tailed *t* test was used for the statistical analysis. n=5 independent cell cultures. (**E**) EdU staining for the assessment of cell proliferation in cardiac fibroblasts overexpressing SNHG7 in the presence or absence of miR-34-5p mimics. Scale bars represented 50 μm. (**F**) Representative images of immunofluorescence staining showing that forced expression of SNHG7-induced fibroblast-myofibroblast transition. Scale bars represent 50 μm. **P<0.05.

To further explore whether SNHG7 promotes cardiac fibrosis via sponging miR-34-5p, the expression level of miR-34-5p in both cardiac tissue and cardiac fibroblasts was determined. As shown in Figure 4A, 4B, the expression level of miR-34-5p was decreased in the infarct area and the peri-infarct areas of cardiac tissues and cardiac fibroblasts treated with TGF-β, which was widely used to construct cell model of fibrosis. In addition, the mRNA levels of Col1α1 and Col3α1 were found to be increased in cardiac fibroblasts transfected with SNHG7. However, the effect was not observed in the cardiac fibroblasts co-transfected by SNHG7 and miR-34-5p(20 nM) (Figure 4C). MTT assay was also performed to explore the effect of SNHG on cell viability. Overexpression of SNHG7 improved the viability of cardiac fibroblasts and co-transfection with miR-34-5p reversed the positive effect of SNHG7 (Figure 4D). The effect of SNHG7 on cardiac fibroblasts proliferation and fibroblast-myofibroblast transition was evaluated to explore the mechanism of SNHG7 in cardiac fibrosis. Results showed that overexpression of SNHG7 accelerated proliferation of cardiac fibroblasts and promoted fibroblast-myofibroblast transition. In contrast, these positive regulatory effects were not noted with the co-transfection of SNHG7 and miR-34-5p (Figure 4E, 4F). Collectively, these data indicated that lncRNA SNHG7 promoted cardiac fibrosis via acting as a ceRNA by targeting miR-34-5p (Figure 4).

### Silencing of lncRNA SNHG7 alleviated TGF-β1 induced fibrogenesis in cardiac fibroblasts

**Figure 5:**
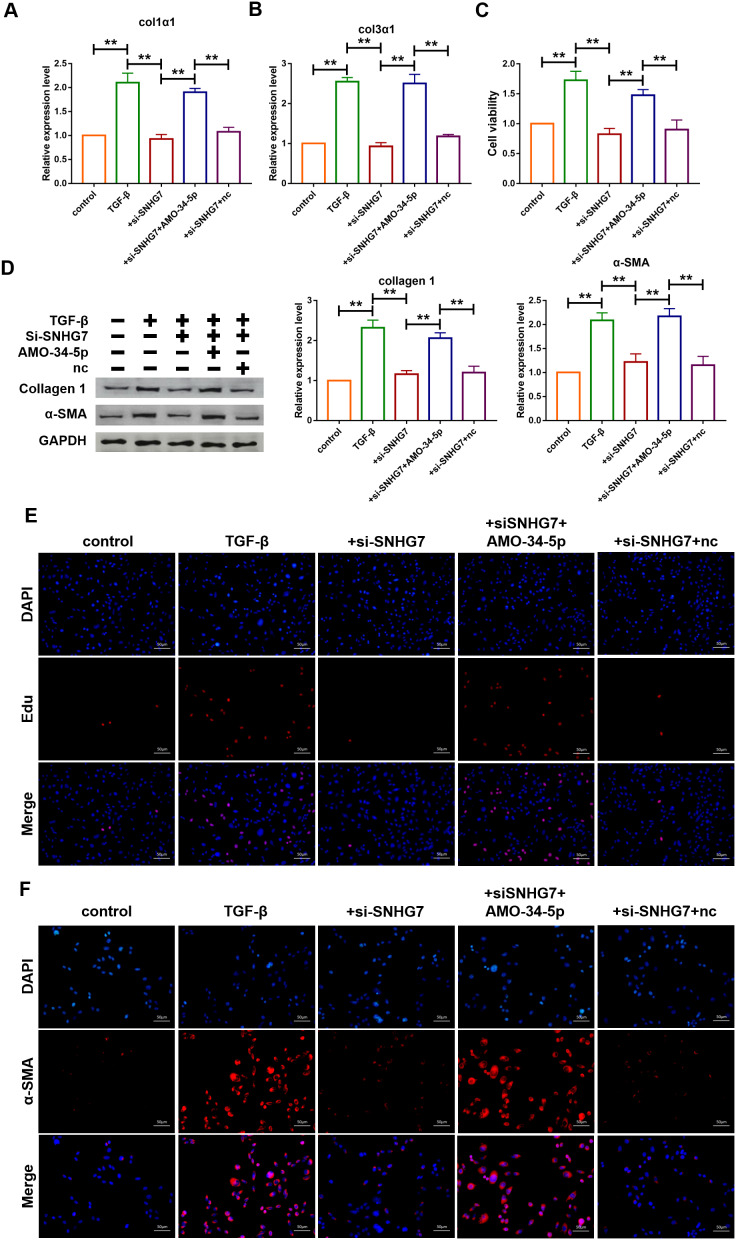
**Silencing of lncRNA SNHG7 alleviated TGF-β1-induced fibrogenesis in cardiac fibroblasts.** (**A**, **B**) Suppression of SNHG7 attenuated the increase in collagen 1^α^1 and collagen 3^α^1 expression induced by TGF-β1, as measured by qRT-PCR; GAPDH mRNA served as an internal control. Data was presented as mean ± SEM; one-way ANOVA was used for the statistical analysis. n=5 independent cell cultures. (**C**) Western blotting analysis showing that knockdown of SNHG7 attenuated TGF-β1-induced fibrotic protein expression (Collagen I and α-SMA); GAPDH served as a loading control. Data was presented as mean ± SEM; one-way ANOVA was used for the statistical analysis. n=5 independent cell cultures. (**D**) MTT assay for the assessment of cell viability. Transfection of si-SNHG7 with or without AMO-34-5p in cardiac fibroblasts treated with TGF-β1 for 24h. Data was presented as mean ± SEM; one-way ANOVA was used for the statistical analysis. n=5 independent cell cultures. (**E**) EdU staining for the assessment of cell proliferation in cardiac fibroblasts inhibiting SNHG7 in the presence or absence of AMO-34-5p mimics. Scale bars represented 50 μm. (**F**) Representative images of immunofluorescence staining showing that knockdown of SNHG7 abated the TGF-β1-induced fibroblast-myofibroblast transition, which was promoted by AMO-34-5p. Scale bars represented 50 μm. **P<0.05.

The role of SNHG7 on fibrogenesis was explored using qRT-PCR analysis and the results showed that transfection of si-SNHG7 significantly inhibited the mRNA expression levels of Col1α1 and Col3α1 (Figure 5A, 5B). In contrast, the inhibition was recovered by the co-transfection of AMO-34-5-p (20 nM). MTT assay was performed to determine cell viability of cardiac fibroblasts. Compared with the TGF-β1 treatment group, inhibition of SNHG7 reduced cell viability of cardiac fibroblasts (Figure 5C). Knockdown of miR-34-5p by AMO-34-5p was found to reversely increase cell viability of cardiac fibroblasts. In addition, western blotting analysis also showed that silencing of SNHG7 suppressed the expression levels of Col1α1 and α-SMA, which could be abolished by treatment with AMO-34-5p (Figure 5D). Cell proliferation and fibroblast-myofibroblast transition were detected to collect further evidence of SNHG7 effect on fibrogenesis. As shown in Figure 5E, knockdown of SNHG7 inhibited the TGF-β1 induced proliferation of cardiac fibroblasts. However, co-transfection with AMO-34-5p was found to promote the proliferation of cardiac fibroblasts. Moreover, the fibroblast-myofibroblast transition induced by treatment of TGF-β1 was diminished by SNHG7 silencing and recovered by co-transfection of AMO-34-5p (Figure 5F). These data indicated that silencing of SNHG7 could alleviate TGF-β1 induced fibrogenesis in cardiac fibroblasts (Figure 5).

### Forced expression of miR-34-5p attenuated cardiac fibrosis induced by myocardial infarction in mice

**Figure 6:**
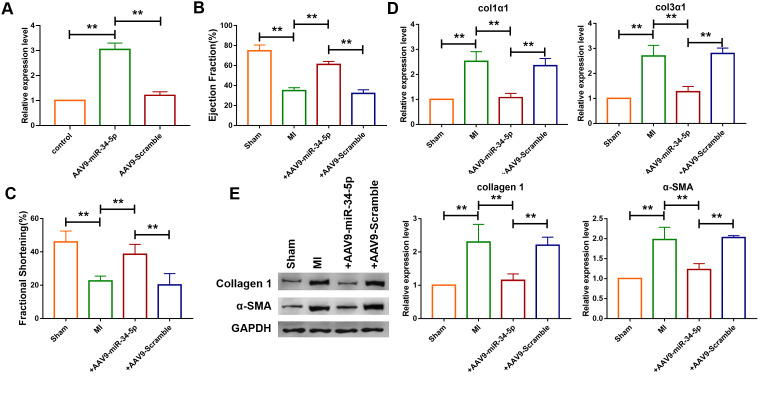
**Overexpression of miR-34-5p ameliorated cardiac fibrosis in the mice after MI.** (**A**) Intravenous injection of AAV9-miR-34-5p via tail increased miR-34-5p expression in normal mice, as measured by qRT-PCR; GAPDH served as an internal control, and AAV9-scramble served as a negative control. Data was presented as mean ± SEM; two-tailed *t* test was used for the statistical analysis. n=5 independent cell cultures. (**B**, **C**) Four weeks after MI, echocardiographic imaging showed that the overexpression of miR-34-5p improved ejection fraction (EF) and fraction shortening (FS). Data was presented as mean ± SEM; one-way ANOVA was used for the statistical analysis. n=12 mice per group. (**D**) qRT-PCR analysis showing that AAV9-miR-34-5p injection reversed the up-regulation of collagen 1α1 and collagen 3α1 in MI mice; GAPDH mRNA served as an internal control, and AAV9-scramble served as an additional control. Data was presented as mean ± SEM; one-way ANOVA was used for the statistical analysis. n=6 mice per group. (**E**) Protein levels of collagen 1 and α-SMA were measured by western blot; GAPDH served as an internal control. Data was presented as mean ± SEM; one-way ANOVA was used for the statistical analysis. n=6 mice per group. **P<0.05.

The mechanism of miR-34-5p in vivo in mice was further investigated. Recombinant adeno-associated virus 9 carrying miR-34-5p precursor (AAV9-miR-34-5p) was initially constructed and injected into mice to overexpress the level of miR-34-5p. As shown in Figure 6A, administration of AAV9-miR-34-5p significantly increased the expression level of miR-34-5p. Echocardiographic findings showed that overexpression of miR-34-5p improved the EF and FS in mice with MI (Figure 6B, 6C). In addition, qRT-PCR analysis demonstrated that upregulation of Col1α1 and Col3α1 levels induced by MI were inhibited by the administration of AAV9-miR-34-5p (Figure 6D). Western blotting analysis revealed that forced expression of miR-34-5p reduced protein levels of Col 1 and α-SMA which were increased in mice with MI (Figure 6E).

### miR-34-5p played cardioprotective role via targeting ROCK1

**Figure 7:**
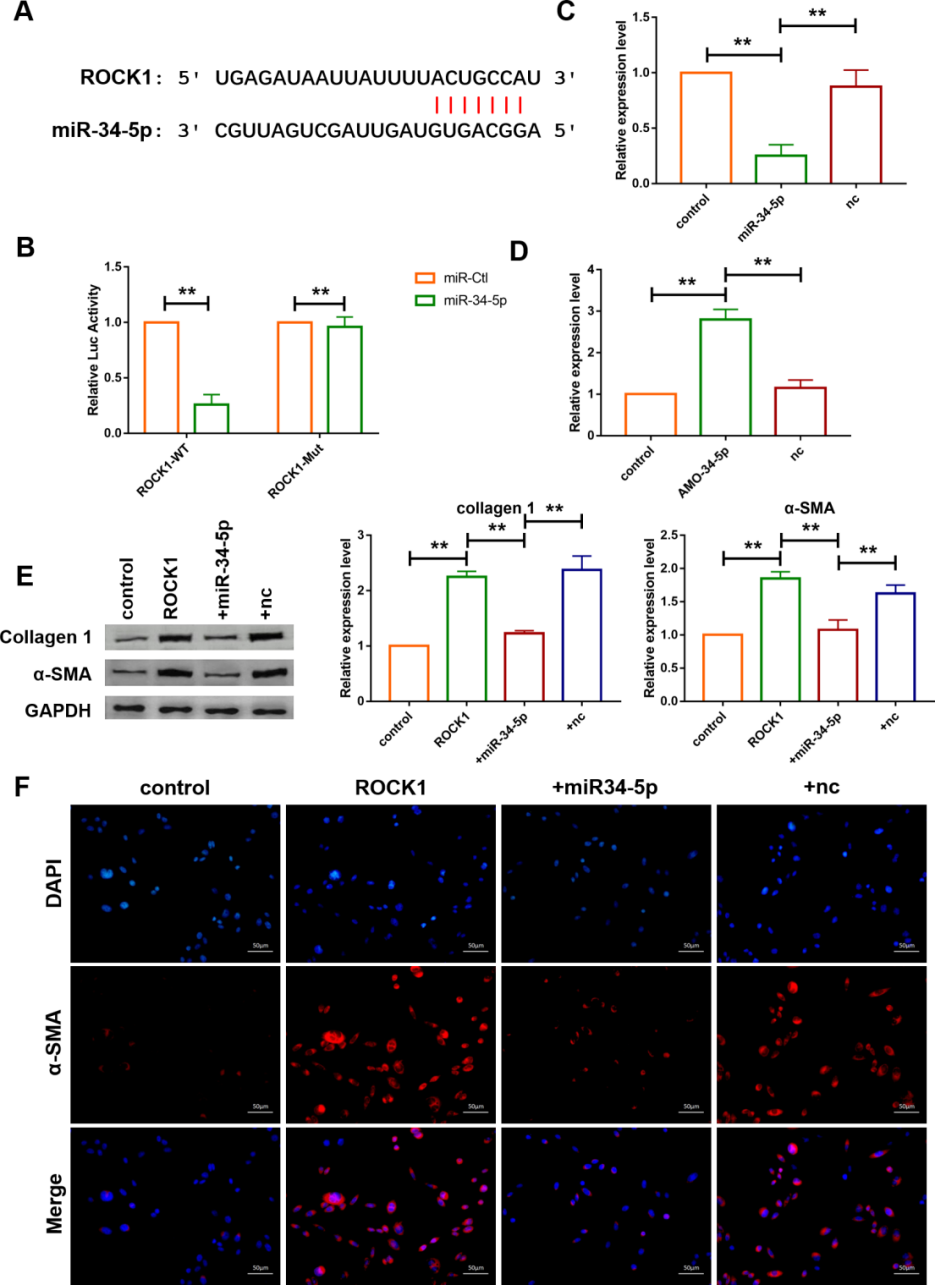
**ROCK1 was a direct target of miR-34-5p and mediated the anti-fibrotic function of miR-34-5p.** (**A**) The predicted binding sites of ROCK1 and miR-34-5p. (**B**) Luciferase reporter activities of chimeric vectors carrying the luciferase gene and a fragment of the 3’ UTR of ROCK1 containing the wild type or mutant miR-34-5p binding sites. Data was presented as mean ± SEM; two-tailed *t* test was used for the statistical analysis. n=5 independent cell cultures. (**C**, **D**) qRT-PCR analysis showing that overexpression of miR-34-5p inhibited the mRNA level of ROCK1 and AMO-34-5p transfection elevated the mRNA level of ROCK1; GAPDH mRNA served as an internal control. Data was presented as mean ± SEM; one-way ANOVA was used for the statistical analysis. n=5 independent cell cultures. (**E**) Protein levels of collagen 1 and α-SMA were measured by western blotting; GAPDH served as an internal control. Data was presented as mean ± SEM; one-way ANOVA was used for the statistical analysis. n=6 independent cell cultures. (**F**) Representative images of immunofluorescence staining showing that overexpression of miR-34-5p diminished fibroblast-myofibroblast transition induced by forced expression of ROCK1. Scale bars represented 50 μm. **P<0.05.

Targetscan database was utilized to further detect the target gene of miR-34-5p, and it was found that there are perfect binding sites of miR-34-5p on the 3’UTR of ROCK1 gene sequence (Figure 7A). Subsequently, luciferase reporter assay was used to examine the interaction between miR-34-5p and ROCK1. As shown in Figure 7B, miR-34-5p, which had no effect on the vector carrying mutated binding site (ROCK1-Mut), inhibited the activity of wild-type ROCK1 (ROCK1-WT) luciferase reporter vector (Figure 7B). Loss-of-function and gain-of-function approaches were utilize to further explore the role of ROCK1 as the target gene of miR-34-5p in cardiac fibroblasts. The result showed that miR-34-5p mimics decreased the expression of ROCK1 and transfection of AMO-34-5p significantly elevated the protein expression level of ROCK1 (Figure 7C, 7D). In addition, western blotting analysis indicated that overexpression of ROCK1 increased the protein expression levels of Col 1 and α-SMA and the effects were diminished by forced expression of miR-34-5p (Figure 7E). Fibroblast-myofibroblast transition was also detected (Figure 7F), and miR-34-5p was found to diminish the promotive effect of ROCK1 on fibroblast-myofibroblast transition of cardiac fibroblasts. These data suggested that the anti-fibrosis function of miR-34-5p was mediated by targeting ROCK1 signaling pathway (Figure 7).

## DISCUSSION

The cardiac fibrosis is characterized by proliferation of cardiac fibroblasts as well as overexpression and secretion of collagens which lead to net accumulation of extracellular matrix proteins in cardiac interstitium. Cardiac fibrosis will inevitably result in diastolic and systolic dysfunction in many cardiac pathophysiologic conditions. Due to regeneration capacity of the myocardium of adult mammals, the most extensive fibrotic ventricular remodeling is always seen in myocardial ischemic injuries, such as myocardial infarction. After the occurrence of MI, collagen-based scar would replace the infarcted myocardium resulted from sudden massive loss of cardiomyocytes. The cellular effectors of cardiac fibrosis have been extensively studied. Cardiac injury leads to changes in extracellular matrix (ECM) of the heart. Secretion of cytokines and growth factors as well as the increased level of mechanical stress jointly control fibroblast phenotype in a dynamic way [26]. TGF-β signaling pathway is the key regulator of cardiac fibrosis. On one hand, the initial activation of TGF-β promotes the expression of collagen and regulates the proliferation and metastasis of fibroblasts; on the other hand, TGF-β pathway enhances the transcription of α-SMA [28]. ROCK1 is a crucial effector involved in the TGF-β signaling pathway and it mediates the downstream regulation of RhoA. Disruption of ROCK1 gene has been proven to attenuate cardiac fibrosis [6, 27, 28], but the upstream regulative mechanism of ROCK1 gene still awaits investigation. Based on the findings of this study, we concluded that ROCK1 was a target of miR-34-5p. Overexpression of miR-34-5p inhibited the expression of mRNA of ROCK1 and knockdown of miR-34-5p significantly elevated the mRNA level of ROCK1. Upregulation of ROCK1 promoted the fibroblast-myofibroblast transition as well as the protein levels of collagen 1 and α-SMA. Co-transfection of miR-34-5p mimics abolished the effect of ROCK1 on fibrogenesis.

MicroRNAs (miRNAs) are a class of short (18-25 nucleotides), endogenous non-coding single-stranded RNA molecules found in eukaryotic cells in recent years. MiRNAs can specifically recognize the 3’UTR of target gene mRNA and inhibit the degradation or translation of target gene mRNA, leading to down-regulation of target gene expression, and these molecules are involved in many biological processes such as cell proliferation, differentiation, apoptosis and disease development. In particular, miRNAs is a crucial regulator in the pathogenesis of cardiac fibrosis induced by ischemic injury. As a member of this family, miR-125b plays a vital role in fibroblast-to-myofibroblast transition [29]. Studies have demonstrated that expression of miR-433 was upregulated in the cardiac fibrosis model and inhibition of miR-433 could ameliorate cardiac remodeling [30] and down-regulation of let-7d contributed to cardiac fibrosis [31]. In this study, we demonstrated the role of miR-34-5p as a new regulative miRNA of cardiac fibrosis, as we have noted that forced expression of miR-34-5p significantly inhibited the proliferation and cell viability of cardiac fibroblasts, whereas overexpression of miR-34-5p using AAV9-miR-34-5p attenuated the collagen production and improved cardiac function in mice after MI.

Increasing evidence have indicated lncRNAs’ involvement in the pathogenesis of many diseases, including fibrosis [32–34]. Previous studies have showed that lncRNA PFAL contributed to the progression of pulmonary fibrosis [35]. Disruption of lncRNA H19 was associated with attenuation of hepatic fibrosis [36]. Despite of the discovery of silencing of lncRNA TCONS_00088786 could inhibit renal fibrosis [37], the mechanism of lncRNA in cardiac fibrosis is still poorly understood. In this study, we found that the expression level of lncRNA SNHG7 was elevated in MI induced cardiac fibrosis in mice for the first time. SNHG7 has previously been reported to play a critical role in carcinogenesis of many tumors. We found that silencing of SNHG7 diminished the development of cardiac fibrosis and improved cardiac function in mice after MI. Collagen deposition was also a critical indicator of fibrosis. In this study, western blotting and qRT-PCR analysis showed that inhibition of SNHG7 decreased the production of collagen. In addition, we further evaluated the mechanism of SNHG7 in pathogenesis of cardiac fibroblasts and MTT assay indicated that SNHG7 increased the cell viability of cardiac fibroblasts and silencing of SNHG7 inhibited cell fibrogenesis and attenuated fibrosis in vitro. All these results highlighted the critical role of SNHG7 in cardiac fibrosis and it may be a novel target for predicting and treating of cardiac fibrosis.

Numerous studies have demonstrated the role of lncRNA as ceRNA by binding to and sponging miRNA [38–40]. During further exploration of the mechanism of SNHG7 in the regulation of cardiac fibrosis, we found that SNHG7 acted as a ceRNA of miR-34-5p, at least in part, in mediating cardiac fibrosis. Luciferase reporter assay indicated that miR-34-5p was one of the targets of SNHG7. And overexpression of miR-34-5p significantly reversed SNHG7-induced cardiac fibroblast proliferation and fibroblast-myofibroblast transition and inhibited the cell viability of cardiac fibroblasts.

In summary, we discovered that lncRNA SNHG7 promoted cardiac fibrosis via targeting miR-34-5p by acting as a ceRNA in mice after MI; silencing of SNHG7 attenuated the deposition of collagen and improved heart function and miR-34-5p inhibited the fibrogenesis of cardiac fibroblasts by targeting ROCK1 and abolished cardiac fibroblasts proliferation and fibroblast-myofibroblast transition that were induced by SNHG7.

## MATERIALS AND METHODS

### Animals

The experiments were conducted in accordance with the previously described protocols and applicable guidelines stipulated by the National Institutes of Health. This study was reviewed and approved by the Institutional Animal Care and Use Committee (IACUC) of Peking University School of Clinical Medicine. C57BL/6 male mice were provided by Cyagen Biosciences (Suzhou, China) and randomly divided into the following groups: Sham group (control; sham-operated), MI (MI model after 4 weeks of left anterior descending artery [LAD] occlusion), MI +AAV9-sh-SNHG7 group, MI + AAV9-sh-scramble group, MI + AAV9-miR-34-5p group and MI + AAV9-scramble group. The construction of MI model was as follows. Briefly, mice were anesthetized by intraperitoneal (i.p.) injection of avertin (2-2-2 tribromoethanol; Sigma-Aldrich) and placed in a supine position on a heating pad (37°C). Mice were intubated with a 19G needle and ventilated with room air via a MiniVent (Type 845) mouse ventilator (Hugo Sachs Elektronik-Harvard Apparatus, Germany). MI was established by permanent ligation of the left anterior descending artery (LAD) using a 7-0 prolene suture. The mice in the sham group served as surgical controls and were subjected to the same procedures as MI mice except for the LAD ligation.

Animals were treated with adeno-associated virus 9 (AAV9) carrying short hairpin RNA for SNHG7 (AAV5-sh-SNHG7) and AAV9 carrying miR-34-5p precursor (AAV9-miR-34-5p) (Biowit Technology, Shenzhen, China) via intra-tracheal injection at a dose of 1x10^11 viral genome (vg) per mouse 7 days before MI. We used AAV9-miR-34-5p to overexpress miR-34-5p and AAV9-sh-SNHG7 to inhibite the expression of lncRNA SNHG7 in vivo.

All animals were kept in Specific pathogen free (SPF) animal rooms (light/dark cycle: 12h/12h; temperature: 20-26°C) with IVC rearing cage. Number of cage companions were 2 and the bedding materials were purchased from Cyagen Biosciences (Suzhou, China) and autoclaved.

All the animals were sacrificed by excessive anesthesia. They were intraperitoneally injected with 3% pentobarbital sodium at a dose of 90 mL/kg. Then the tissues in the left ventricle were collected for biochemical assay. And the whole hearts were excised and immediately snap frozen for Masson’s staining.

### Cell culture

Cardiac fibroblasts were isolated from the hearts of newborn C57BL/6 mice with trypsin and the procedures were as follows. After newborn C57BL/6 mice were disinfected in 75% alcohol and decapitated, the hearts were aseptically excised with scissors. Then the hearts were cut into small pieces and placed in Dulbecco’s Modified Eagle Media (DMEM, HyClone, UT) without fetal bovine serum (FBS) and then the tissues were digested in 0.25% trypsin solution (Beyotime Biotechnology, Beijing). The digested cell suspensions were centrifuged at 1000 rpm for 5 min and the pellet was re-suspended in DMEM medium containing 8% FBS for 2 h. Then cardiomyocytes were purified through differential adhesion and stored for use in other experiments and the remained adhesive cells were cardiac fibroblasts. New fresh culture media (10% FBS) were added and the fibroblasts were cultured in an5% CO2 incubator for 24 h.

### Cell transfection

For overexpression of SNHG7, full-length SNHG7 was amplified by PCR and subcloned into pcDNA3.1, and stable clones were obtained by G418 screening. Empty vector of pcDNA3.1 was used as the negative control. For knockdown of SNHG7, the negative controls (nc) of siRNA targeting SNHG7 and a scrambled siRNA were synthesized. All plasmids were isolated using AxyPrep DNA Miniprep Kit (Axygen, USA). The miR-34-5p mimics, inhibitors, and respective negative controls were obtained from RiboBio (Guangzhou, China). HEK293 and cardiac fibroblasts were transfected with X-treme GENE siRNA transfection reagent (Roche, Germany) for 6 h according to the instructions provided by the manufacturer. Then the transfection medium was changed to culture medium with 10% FBS. After transfection for 24h, cardiac fibroblasts were treated with TGF-β (10ng/ml) for 24 h. Efficiencies of siRNA and expression plasmid, mimics and inhibitor were determined (Supplementary Figure 1).

### Quantitative real time polymerase chain reaction (qRT-PCR)

Total RNA was isolated from tissue samples and cells in accordance with the standard protocol. And then, the purity and concentration of RNA was determined and all the samples were converted into cDNA using reverse transcription kit. qRT-PCR was performed using the SYBR Green (Thermo Fisher Scientific) system and the data was analyzed using GraphPad 7.

Western blotting analysis Protein samples were blotted onto nitrocellulose membranes in accordance with the standard protocol and the membranes were scanned using the Odyssey Infrared Scanning System (Gene Co. Ltd., Hongkong, China). Finally, the western blotting results were analyzed using the Image J software.

The antibodies used were listed as follows:collagen 1 antibody was produced by Proteintech Group (Wuhan, China), α-SMA antibody by Cell Signaling Technology (Danvers, MA, USA), secondary antibodies IRDye700 (mouse) or IRDye800 (rabbit) by LICOR (Lincoln, Nebraska, USA).

### Luciferase reporter assay

A miR-34-5p sensor reporter was constructed to evaluate the impact of SNHG7 on miR-34-5p. Cardiac fibroblasts were transfected with miR-34-5p sensor and others as described in the corresponding figures. The plasmid of psiCHECK-2 luciferase reporter was inserted with the wild-type or mutant ROCK1-3’UTR sequences that contain the putative binding sites of miR-34-5p. miR-CTL or miR-34-5p mimics were transfected with reporter vectors into endothelial cells. The cells were collected at 48 h post-transfection and lysed to determine luciferase activity using Dual-Luciferase Assay System (Promega, USA).

### Echocardiograph

Mice were anesthetized with inhalation anesthesia using 1% isoflurane-. And cardiac hemodynamics property was evaluated using a Vevo2100 system (VisualSonics, Toronto, Canada) 4 weeks post-MI. Mice were anesthetized and placed in a supine position on a heating pad and two-dimensional M-mode echocardiography with a 30MHZ transducer was used to assess left ventricular function, and fractional shortening (FS) and ejection fraction (EF) were evaluated.

### MTT assay

Cardiac fibroblasts were plated on 96-well plates and MTT assay was used to determine cell viability. After miRNA transfection and TGF-β treatment were performed, MTT (0.5 mg/mL; Beyotime Biotechnology, China) was added to each well and the cells were incubated at 37°C for 3 h. Then 150 μL DMSO was added and the mixed solution was incubated for 15 min. Finally, the absorbance was measured at 493 nm using Spectrophotometer (Tecan, Austria).

### Immunofluorescence staining

Cardiac fibroblasts were plated in 24-well plates. After miRNA transfection and TGF-β treatment, the cells were rinsed with (phosphate buffer saline) PBS buffer and fixed with 4% paraformaldehyde and permeabilized with 0.2% Triton-X-100 solution in PBS. Then the cells were blocked using 10 % goat serum. incubated overnight with α-SMA antibody at 4 °C followed by incubation with fluorescein isothiocyanate (FITC)-conjugated goat anti-mouse antibodies for 1h. Finally, the cells were incubated with 4′,6-diamidino-2-phenylindole (DAPI) after three times of rinsing with PBS.

### EdU assay

Cardiac fibroblasts were seeded in 24-well plates and added with EdU solution (Ribobio, China) on accordance with the protocols. The cells were incubated for 2 h, and then treated and photographed.

### Statistical analysis

The number of independent experimental replications and precisions measurements were presented in the corresponding figures. All data was presented as a mean ± S.E.M. Statistical analyses were performed using the GraphPad Prism 7 software and assessed by the two-tailed Student’s t test or a one-way ANOVA. P < 0.01 was considered as statistically significant.

## Supplementary Material

Supplementary Figure 1
