# Supplementary material for: LncRNA SNHG7 promotes cardiac remodeling by upregulating ROCK1 via sponging miR-34-5p
Source: Aging (Albany NY). 2020 Jun 6;12(11):10441–56. doi: 10.18632/aging.103269 (PMC7346013; doi:10.18632/aging.103269)
Supplement: Supplementary Figure 1 [file aging-12-103269-s001..pdf]

## SUPPLEMENTARY FIGURE

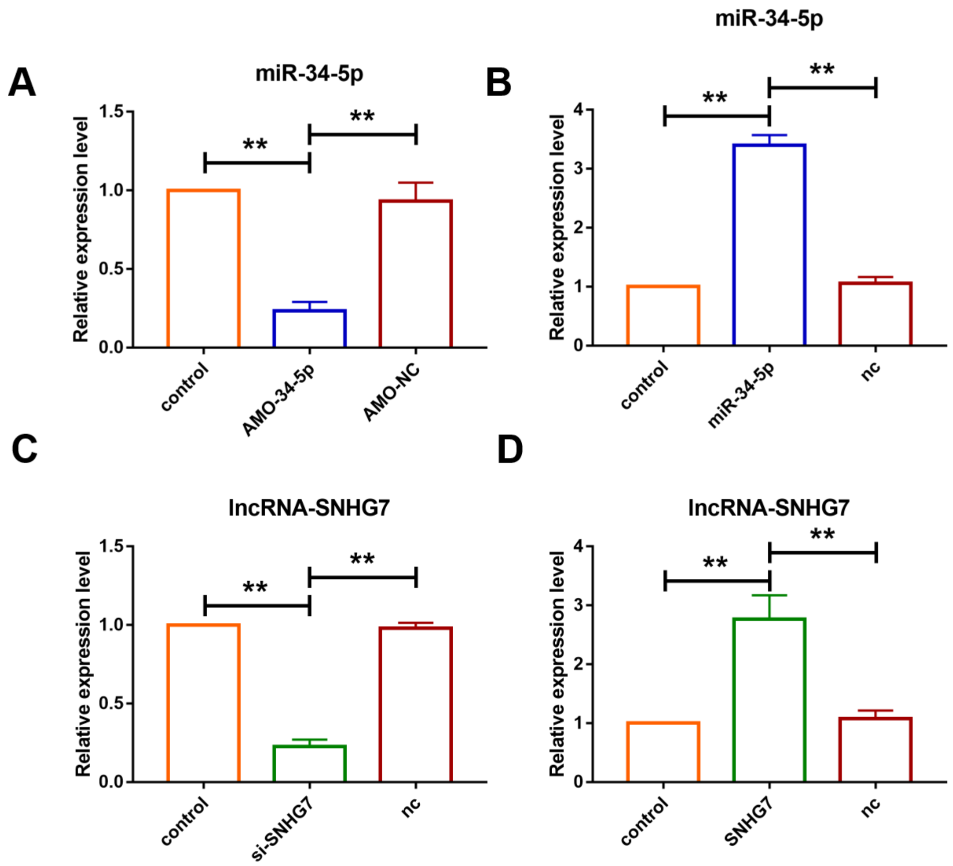


**Supplementary Figure 1. Determination of efficiencies of siRNA and expression plasmid, mimics and inhibitor.** (**A**–**D**) qRT-PCR analysis showing that overexpression and inhibition of miR-34-5p and lncRNA SNHG7. GAPDH mRNA served as an internal control. Data was presented as mean ± SEM; one-way ANOVA was used for the statistical analysis. n=5 independent cell cultures. ***P*<0.05.
